# Trauma-Triggered Psychotic Episodes in Post-traumatic Stress Disorder: A Case Report

**DOI:** 10.7759/cureus.100017

**Published:** 2025-12-24

**Authors:** Rafael Carvalho, Emanuel Santos

**Affiliations:** 1 Psychiatry, Unidade Local de Saúde de Santo António - Hospital de Magalhães Lemos, Porto, PRT

**Keywords:** brief psychotic episodes, complex ptsd, intimate partner violence, psychosis and trauma, ptsd, reproductive coercion, trauma reactivation

## Abstract

Post-traumatic stress disorder (PTSD) is a clinically complex condition, and its intrinsically intense emotional burden may, in some cases, be associated with trauma reactivation that precipitates transient psychotic symptoms, particularly in individuals exposed to chronic interpersonal trauma. Although psychosis is not part of PTSD diagnostic criteria, trauma-related psychotic experiences have been documented and may emerge in the context of hyperarousal, intrusive recollections, and perceived threat amplification. We present the case of a 46-year-old woman with PTSD following prolonged intimate partner violence who experienced three brief psychotic episodes, each triggered by emotionally salient trauma cues: a difficult separation following an abusive relationship, the postpartum period marked by the reactivation of memories of reproductive coercion, and an unexpected encounter with her former abuser. The most recent episode involved acute persecutory delusions centered on digital surveillance, accompanied by intense anxiety and hyperarousal, with partial insight. Symptoms resolved fully within weeks after resuming psychological and psychiatric follow-up, including antipsychotic treatment. Notably, each episode was separated by several years of complete remission, with the patient maintaining stable functioning even after discontinuation of antipsychotic therapy.

This case illustrates how trauma triggers can be associated with brief psychotic experiences in susceptible individuals and underscores the importance of recognizing trauma-related mechanisms to avoid misdiagnosis with primary psychotic disorders. Early identification, psychoeducation, supportive and trauma-focused interventions, and short-term antipsychotic therapy can lead to rapid resolution and sustained stability.

## Introduction

Post-traumatic stress disorder (PTSD) is characterized by intrusive re-experiencing, avoidance of reminders, negative alterations in cognition and mood, and marked arousal and reactivity following exposure to trauma [1]. Symptoms must persist for at least one month and may follow a chronic course, particularly in the context of prolonged interpersonal trauma. In recent years, the concept of complex PTSD (cPTSD), as defined in the International Classification of Diseases, 11th Revision (ICD-11), has gained increasing recognition, encompassing not only core PTSD symptoms but also disturbances in self-organization, including affect dysregulation, persistent negative self-concept, and interpersonal difficulties, commonly associated with prolonged or repeated interpersonal trauma, such as long-term coercive control or domestic violence [2].

Although psychotic symptoms are not part of the formal diagnostic criteria for PTSD, an increasing body of research demonstrates strong associations between PTSD, especially when involving cPTSD symptomatology, and psychotic experiences [3-6]. Brief psychotic episodes triggered by trauma reminders often mirror the thematic content of the original trauma, and the association between trauma exposure and psychotic experiences may be mediated by PTSD symptoms [7]. Cumulative trauma exposure, including chronic coercive control or domestic violence, is associated with heightened vulnerability to both PTSD and psychotic symptoms [8,9]. The postpartum period represents an additional window of vulnerability, which may intensify trauma reactivation and destabilize emotional regulation [10]. Understanding the temporal link between trauma cues, autonomic hyperarousal, and the emergence of paranoid ideation is essential to avoid misdiagnosis with primary psychotic disorders [11]. By contrast, schizophrenia-spectrum disorders are usually characterized by a persistent or progressive course, with incomplete inter-episodic remission and cumulative functional impairment. Moreover, early recognition of trauma-related psychotic symptoms is crucial, as trauma-focused interventions can be implemented in patients with comorbid psychotic symptoms when appropriately monitored [12].

We present a case of a patient with PTSD related to prolonged domestic violence exposure, who experienced three brief psychotic episodes, each triggered by trauma-related cues and resolving completely with treatment. The case highlights the phenomenological overlap between trauma-related hyperarousal, re-experiencing, and transient psychotic experiences, as well as the importance of contextualizing psychotic symptoms within the broader framework of trauma.

## Case presentation

The present case concerns a 46-year-old married woman living with her husband and two daughters, who presented to a psychiatric consultation for follow-up. She first engaged in psychiatric care approximately a decade earlier, following the dissolution of her first marriage, which had been characterized by prolonged intimate partner violence, including a pattern of coercive control with psychological abuse, verbal intimidation, reproductive coercion, and episodes of sexual abuse, occurring over several years during her first marriage. In the months following the separation, she developed symptoms consistent with PTSD, including vivid intrusive memories and recurrent nightmares related to episodes of abuse, intermittent flashbacks, intense psychological distress when exposed to trauma reminders, persistent avoidance behaviors, and marked hypervigilance, lasting several months and causing significant impairment in familial, occupational, and social functioning. She also reported feeling persecuted and monitored by her ex-husband at that time, experiences that, in an early phase, appeared to be embedded in a trauma-related fear response. These symptoms caused significant impairment in her familial, occupational, and social functioning and led to psychiatric and psychological treatment, with substantial improvement over time. She denied any history of substance use, developmental disorders, obsessive-compulsive symptoms, symptoms suggestive of primary mood or psychotic disorders, suicidal ideation, or self-harm behaviors, and she had never been hospitalized for psychiatric reasons. She also denied any relevant family psychiatric history, including psychotic or bipolar disorders.

Over the following years, she experienced three brief psychotic episodes, all of them characterized by acute-onset psychotic symptoms lasting less than one month, with full remission, and each closely linked to periods of increased vulnerability and trauma reactivation. The first episode occurred following the aforementioned onset of PTSD symptoms, shortly after the divorce of her first and reportedly offending husband. The second episode emerged four years later, after remarriage, during the postpartum period of her first child, a time marked by social isolation and increased emotional vulnerability and the resurgence of traumatic memories specifically connected to her former husband, who had previously attempted to coerce her into having children against her will. The transition to motherhood triggered vivid recollections of this reproductive coercion, accompanied by intrusive memories, nightmares, and heightened physiological arousal. The most recent episode developed after unexpectedly seeing her former husband in public, following several years of clinical remission. Each episode was characterized by the sudden onset of persecutory ideas and prominent anxiety, but no sustained mood changes or hallucinatory experiences were reported. Those episodes were characterized by a lack of full insight, with intermittent awareness of being unwell, without a consistent capacity to question the core belief.

During the most recent presentation, she reported a firm belief held with delusional conviction that her former husband had resumed persecutory behavior, primarily through digital intrusion into her devices, online accounts, and videoconferencing platforms. She was convinced that he had gained access to her computer, personal accounts, and even her online meetings, leading to increased hypervigilance and repetitive checking behaviors. Although she occasionally noted that some thoughts might be exaggerated, these moments were fleeting, and the predominant clinical picture was one of strong conviction in the core persecutory delusion. No dissociative symptoms, such as depersonalization, derealization, or dissociative amnesia, were observed or reported. She had a lack of full insight, characterized by intermittent awareness of being unwell, primarily attributed to intense anxiety and hyperarousal. Her husband reported that she had been increasingly preoccupied and distressed by these suspicions over several weeks, even when he repeatedly confronted her with the absence of objective evidence. On mental status examination, she appeared well-groomed and cooperative, with an anxious mood, thought content dominated by persecutory delusions without hallucinations or marked formal thought disorder, and lacking full insight.

She was started on paliperidone 6 mg, resumed psychological follow-up, and within two weeks, her psychotic symptoms resolved completely. She later reported that her digital concerns were clarified after a technical review identified and removed malware from her computer. She regained full insight, resumed her usual occupational and family activities, and did not require any further adjustment in medication.

**Figure 1:**
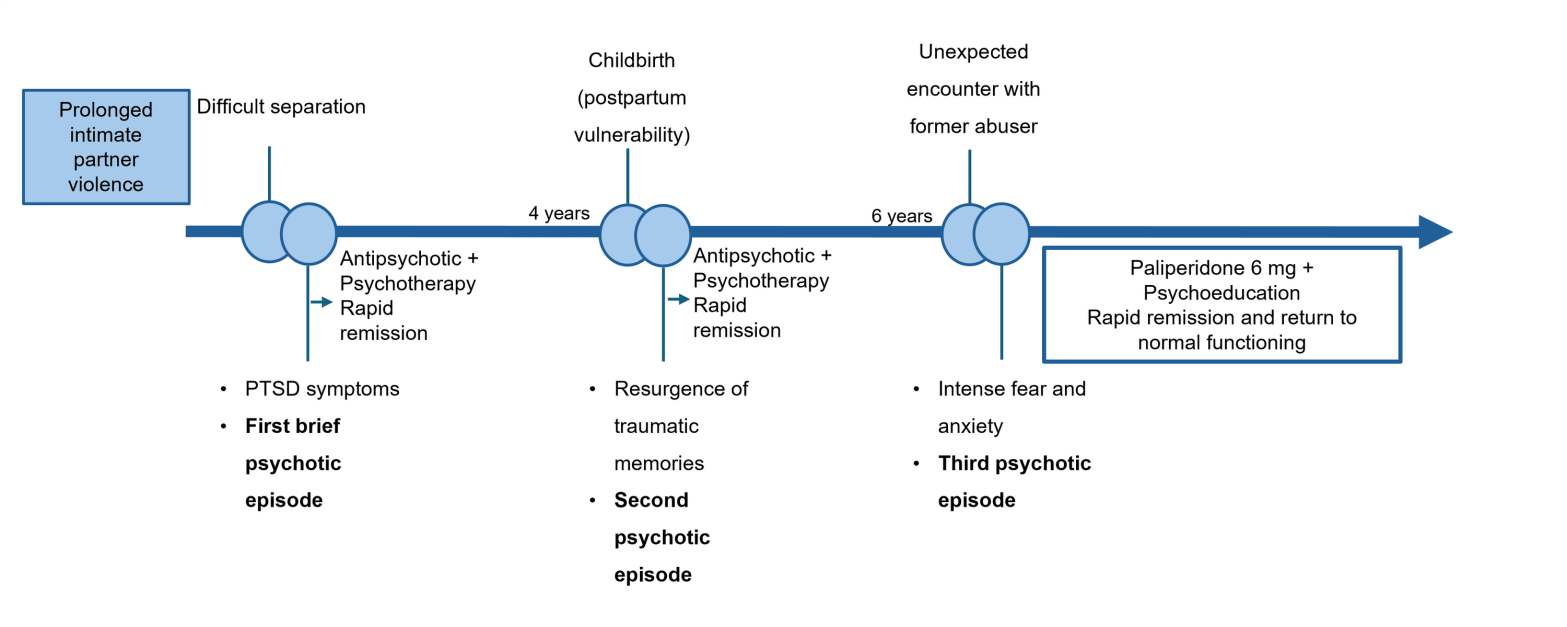
Clinical timeline summarizing major traumatic events, psychotic episodes, and treatment interventions. PTSD: post-traumatic stress disorder

She consistently demonstrated excellent functioning between episodes, maintaining stable employment, fulfilling social and familial roles, and enjoying a supportive relationship with her current husband and daughters. In both previous episodes, she had been treated with a low-to-moderate dose antipsychotic with rapid remission occurring within weeks and treatment maintained for only a few months. After each remission, antipsychotic medication was gradually discontinued without relapse for several years. During these asymptomatic intervals, she remained under psychiatric and psychological follow-up for residual PTSD symptoms such as hyperarousal and occasional intrusive memories of episodes of physical aggression, and she was intermittently treated with antidepressants or anxiolytics for these symptoms. Over the years, she has remained free from chronic psychosis, with each episode showing rapid and complete remission once treated and prolonged periods of normal functioning thereafter. A summary of the clinical course, major trauma-related events, and treatment interventions is presented in Figure 1.

## Discussion

This case illustrates the complex relationship between trauma, perceived control, and psychotic experiences in a patient exposed to years of coercion and domestic violence. The patient’s psychotic episodes shared a consistent thematic core of persecution, intrusion, and surveillance, echoing the controlling dynamics of her earlier abusive relationship. The delusional content, centered on a violation of personal boundaries, symbolically reflected the earlier trauma of loss of autonomy and invasion of her private space. These experiences did not emerge randomly but were reactivated during periods of heightened vulnerability and emotional stress.

The postpartum episode highlights the role of reproductive coercion as a trauma trigger, consistent with evidence that the perinatal period heightens vulnerability to trauma reactivation and psychiatric instability [10]. The birth of her child served as a potent trigger for memories of her ex-husband’s attempts to force pregnancy, reawakening feelings of fear, loss of autonomy, and bodily intrusion. These trauma-linked cognitive and emotional states contributed to a transient but fully formed psychotic episode, characterized by heightened arousal, intrusive recollections, and paranoid delusions.

The physiological hyperreactivity associated with PTSD, including increased sympathetic activation, heightened threat detection, and impaired modulation of fear circuits, may explain how a single encounter with her former abuser was sufficient to trigger a psychotic experience, consistent with stress-reactivity models of trauma-related psychosis [13]. The intensity of re-experiencing and fear overwhelmed cognitive appraisal, leading to acute disorganization and persecutory interpretations. Importantly, her partial insight, acknowledging that something was not right with her, supports the view that the paranoid experience may thus represent an extreme manifestation of trauma-related hyperalertness and emotional overload rather than a primary psychotic disorder. The patient retained the ability to recognize the connection between her distress, anxiety, and somatic activation, suggesting a degree of metacognitive integration despite the intensity of her persecutory ideas.

The rapid and complete remission of psychotic symptoms following recognition of the episode, re-engagement with psychological and psychiatric support, and the introduction of an antipsychotic reinforces the view that timely intervention and psychoeducation can effectively contain and resolve these acute stress-related psychotic reactions. Psychoeducation played a key role in facilitating insight, reducing anxiety, and promoting rapid engagement with care. In parallel, the patient engaged in cognitive-behavioral psychotherapy over a sustained period, primarily targeting trauma-related symptoms, coping strategies for managing stress and trauma reminders, as well as relapse prevention. The pharmacological component likely contributed to the modulation of dopaminergic hyperactivation secondary to stress, while the therapeutic alliance and supportive environment helped restore a sense of safety, cognitive coherence, and reduce trauma-related arousal.

The absence of dissociative phenomena during episodes further supports their classification as brief psychotic reactions related to acute stress rather than dissociative episodes. The sustained remission of psychotic symptoms achieved through ongoing psychotherapy also points to a favorable long-term prognosis. Her long periods of complete remission, good social functioning, and capacity for emotional closeness contrast sharply with the course expected in schizophrenia-spectrum disorders [11].

## Conclusions

This case reinforces the notion that trauma-related psychosis lies on a continuum with PTSD, particularly when the psychotic content is thematically tied to the trauma and emerges during intense reactivation states. It also highlights how affect dysregulation, shame, and interpersonal withdrawal, features compatible with cPTSD, may appear during trauma-triggered states.

PTSD, particularly when associated with prolonged interpersonal trauma, can manifest with significant heterogeneity. Although psychotic symptoms are not included in diagnostic criteria, this case exemplifies how transient psychotic episodes may arise in close temporal and thematic association with trauma reactivation. The delusional content reflected past experiences of coercion and intrusion, and each episode was triggered by emotionally salient cues. Recognizing such presentations as trauma-related rather than primary psychotic disorders is crucial, as prognosis and management differ significantly. Early identification, psychoeducation, supportive psychotherapy, and, when indicated, short-term antipsychotic treatment can lead to rapid resolution and sustained remission. This case underscores the importance of understanding trauma-related psychosis as a dimensional and context-dependent phenomenon, deeply rooted in the psychological and physiological sequelae of chronic interpersonal trauma.
